# Cross-species functional analyses reveal shared and separate roles for Sox11 in frog primary neurogenesis and mouse cortical neuronal differentiation

**DOI:** 10.1242/bio.015404

**Published:** 2016-03-09

**Authors:** Chao Chen, Jing Jin, Garrett A. Lee, Elena Silva, Maria Donoghue

**Affiliations:** Department of Biology, Georgetown University, 37th and O Street NW, Washington, DC 20057, USA

**Keywords:** Sox transcription factor, Neural development, Neuronal differentiation

## Abstract

A well-functioning brain requires production of the correct number and types of cells during development; cascades of transcription factors are essential for cellular coordination. Sox proteins are transcription factors that affect various processes in the development of the nervous system. Sox11, a member of the SoxC family, is expressed in differentiated neurons and supports neuronal differentiation in several systems. To understand how generalizable the actions of Sox11 are across phylogeny, its function in the development of the frog nervous system and the mouse cerebral cortex were compared. Expression of Sox11 is largely conserved between these species; in the developing frog, Sox11 is expressed in the neural plate, neural tube and throughout the segmented brain, while in the mouse cerebral cortex, Sox11 is expressed in differentiated zones, including the preplate, subplate, marginal zone and cortical plate. In both frog and mouse, data demonstrate that Sox11 supports a role in promoting neuronal differentiation, with Sox11-positive cells expressing pan-neural markers and becoming morphologically complex. However, frog and mouse Sox11 cannot substitute for one another; a functional difference likely reflected in sequence divergence. Thus, Sox11 appears to act similarly in subserving neuronal differentiation but is species-specific in frog neural development and mouse corticogenesis.

## Abstract (precis)

**Summary:** Sox11 acts to designate neurons in both mouse and frog brains, but orthologs are not functionally redundant. These data show evolutionary conservation of Sox11 function with molecular divergence.

## INTRODUCTION

Neurogenesis, the process of generating neurons from progenitor cells, is tightly regulated to produce sufficient numbers of neurons and glia throughout development (Kessler and Melton, 1994; Kageyama et al., 1997; Molyneaux et al., 2007). Either intrinsic programs or environmental signals control neural progenitor proliferation, cell cycle exit, or commitment to a neuronal fate. As progenitors cease division, proneural transcription factors initiate cascades of gene expression that activate genes responsible for neuronal differentiation (Lee et al., 1995; Ma et al., 1996; Kiefer et al., 2005; Wilkinson et al., 2013). As neurons mature, their cell morphology becomes more complex. Eventually, synapses form between neurons that transduce signals within defined circuits (Kriegstein, 2005; Koleske, 2013).

The Sox transcription factors play key roles in multiple steps as neural cell progress to their final fate (Bergsland et al., 2011). The Sox family of transcription factors is composed of approximately 20 members in vertebrates, each of which contains a high mobility group (HMG) DNA binding domain (Bowles et al., 2000). Based upon sequence homology, eight subfamilies, A-H, were defined. Interestingly, members of a single subfamily tend to subserve the same biological functions (Kamachi and Kondoh, 2013). For example, SoxB1 members, Sox1, 2 and 3, maintain neural progenitor pools (Buescher et al., 2002; Bylund et al., 2003; Graham et al., 2003; Rogers et al., 2009), whereas SoxC members, Sox4, 11 and 12, promote neuronal differentiation (Dy et al., 2008; Hoser et al., 2008; Potzner et al., 2010; Mu et al., 2012; Shim et al., 2012; Wang et al., 2013).

The SoxC protein Sox11 acts during neuronal differentiation in several species and regions of the nervous system. Sox11 promotes neuronal differentiation in the chick spinal cord and mouse hippocampus (Bergsland et al., 2006; Mu et al., 2012), directs neuronal specification in the mouse cortex (Lai et al., 2008; Shim et al., 2012; Chen et al., 2015), and enhances axonal growth and survival in mouse sympathetic neurons (Thein et al., 2010; Lin et al., 2011). Sox11 constitutive knockout mice die at birth (Sock et al., 2004), whereas Sox11 conditional knockout mice have fewer neurons in the brain (Chen et al., 2015). Sox11's conserved role in neurogenesis between species, however, remains unclear.

To understand roles for Sox11 in the formation of the nervous system, similarities and differences between expression and function in frog neural tube development and mouse cerebral cortex formation were studied. First, expression of Sox11 in the mouse cerebral cortex was examined, revealing dynamic expression in neurons of the differentiated zones, with activation and then inactivation as corticogenesis proceeds. In addition, gain- and loss-of-function studies demonstrated that Sox11 promotes neuronal differentiation and neurite outgrowth in mouse cortical neurons. Next, the expression pattern and function of *sox11* was examined in frog neural development. *sox11* is expressed in neural tissue and, unlike the pattern observed in mouse, expression persists throughout primary neurogenesis. Functional analysis suggests that Sox11 plays roles in both neural induction, the commitment of ectoderm to neural tissue, and molecular and morphological shifts associated with neuronal differentiation in frog. Interestingly, while pro-differentiation functions were similar between mouse and frog, the Sox11 orthologs could not substitute for one another in experimental paradigms. Bioinformatic analyses of mouse and frog Sox11 sequences highlight a single amino acid difference in the HMG domain as well as significant variation in the sequences outside of this region. Thus, species-specific differences are likely explained by sequence divergence.

## RESULTS

### A Role for Sox11 in mouse corticogenesis

**Fig. 1.**
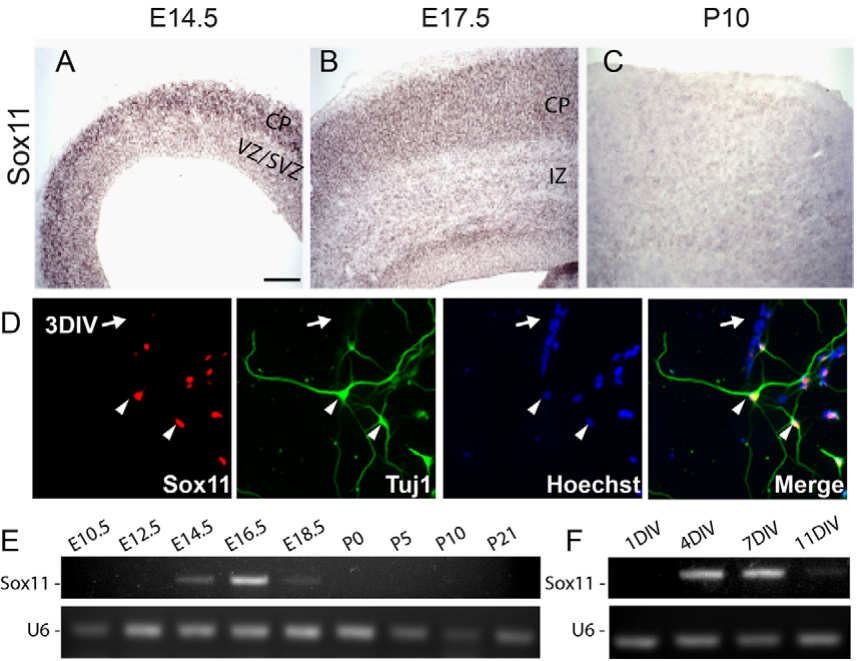
**Sox11 is expressed in differentiated neurons *in vivo* and *in vitro* and is dynamically expressed during mouse corticogenesis.** (A-C) Wild type cerebral cortex hybridized with antisense probes specific for Sox11 at E14.5 (A), E17.5 (B) and P10 (C), with expression visualized using BM Purple. Sox11 is expressed in the cortical plate (CP) at E14.5 and E17.5. There is no detectable expression of Sox11 at P10. (D) In cultured cortical neurons examined after 3 days *in vitro* (3DIV), expression of Sox11 is localized to cells that are TuJ1^+^ (arrowheads) and absent from TuJ1^−^ cells (arrow). (E-F) Profile of Sox11 expression during cortical development *in vivo* (E) and *in vitro* (F) reveals undetectable levels early (E10.5-E12.5 and 1DIV), activation (E14.5-E18.5 and 4DIV), and inactivation (P0-P21 and 11DIV). CP, cortical plate; VZ, ventricular zone; SVZ, sub-ventricular zone; IZ, intermediate zone. Scale bar: 60 μm (A,B); 130 μm (C); 25 μm (D).

To begin this analysis, expression of Sox11 in the mouse cerebral cortex was examined. *In situ* hybridization and immunohistochemistry for Sox11 was performed to characterize localization at different developmental ages (Fig. 1A-D; Fig. S1). Sox11 is preferentially expressed in the subplate, marginal zone, and cortical plate at embryonic day (E)14.5 and E17.5 (Fig. 1A,B; Fig. S1). Postnatally [postnatal day (P)10], Sox11 levels were not detectable (Fig. 1C). Cortical embryonic expression followed by postnatal downregulation was confirmed by RT-PCR analysis of embryonic cortical samples (Fig. 1E). Sox11 was first detectable at E14.5, peaked at E16.5, decreased to low levels at E18.5, and was not detectable at P10 (Fig. 1E).

In addition to regulated expression in intact cerebral cortex, Sox11 is also selectively expressed in cultured primary cortical neurons (Fig. 1D,F). In many ways, the differentiation of cortical neurons grown in culture mimics the developmental progression of neurons in the cortex; newly plated cells from the E14.5 cortex gradually differentiate in culture, becoming more and more mature. Using immunohistochemistry for Sox11 and the neuron-specific anti-Tubb3 antibody, Sox11 was not detectable one day after plating [1 day *in vitro* (1DIV)] (Fig. 1F), but was expressed in TuJ1^+^ (antibody stains for class III β-tubulin, Tubb3) cells by 3DIV (Fig. 1D). RT-PCR analysis revealed that Sox11 expression was low at 1DIV, high at 4DIV and 7DIV, and low again at 11DIV (Fig. 1F). Thus, the same activation and inactivation of Sox11 observed in the cerebral cortex *in vivo* (Fig. 1E) is present in cortical cultures grown *in vitro* (Fig. 1F). Together, these observations demonstrate that Sox11 expression parallels neuronal differentiation in the mouse cortex.

**Fig. 2.**
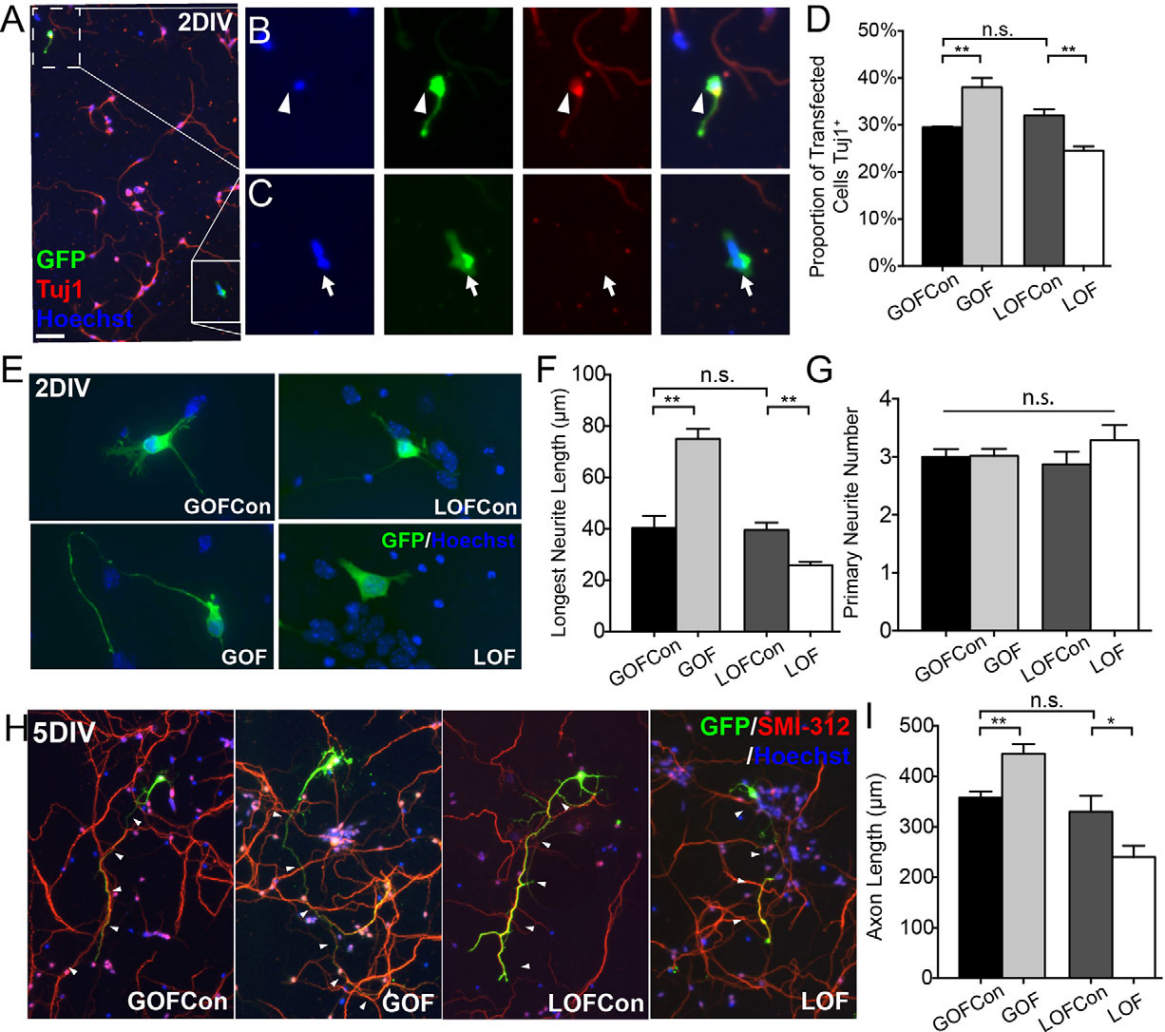
**Perturbation of Sox11 shifts proportions of neurons and alters neuronal morphology of mouse cortical neurons *in vitro*.** (A-C) A representative image of cortical neurons transfected with GFP (green), grown for 2DIV, and stained with TuJ1 (red) and Hoechst (blue) (A). GFP^+^-transfected cells are either TuJ1^+^ neurons (B, arrowhead) or TuJ1^−^ cells (C, arrow). (D) The proportion of transfected cells that are TuJ1^+^ is greater in Sox11GOF and lower in Sox11LOF, compared to appropriate control cultures. (E-G) Cells transfected with control (top), Sox11GOF (bottom left), or Sox11LOF (bottom right), stained for GFP and labeled with Hoechst, and imaged at 2DIV (E). The average length of the longest neurite is longer in Sox11GOF and shorter in Sox11LOF compared to controls (F) whereas there is no difference in the number of primary neurites (G). (H-I) GFP^+^ cells transfected with control vectors (left and third from left), Sox11GOF (second from left), or Sox11LOF (right) were harvested at 5 DIV and stained with SMI-312 (red) to mark axons (H). Average axon length was longer in Sox11GOF and shorter in Sox11LOF compared to control cells (I). Scale bar: 60 μm (A); 24 μm (B,C); 12 μm (E); 52 μm (I). Data represented as mean±sem; n.s., not significant; *P<0.05; **P<0.01.

To examine the function of Sox11 in mouse cortical development, *ex utero* electroporation (EUE) was used to transfect cortical cells with a GFP plasmid and either a control plasmid, a Sox11 expression vector (gain-of-function, GOF), or a Sox11 shRNA vector (loss-of-function, LOF). Following EUE, cortical cells were dissociated and grown under conditions that promote neuronal differentiation. At 2DIV, cultures were stained with TuJ1 to identify neurons and the characteristics of GFP^+^ transfected cells were examined (Fig. 2A-C). Compared to control transfected cells, a greater proportion of the GFP^+^ Sox11GOF cells also expressed Tubb3 and a smaller proportion of GFP^+^ Sox11LOF cells were TuJ1^+^ (Fig. 2D), consistent with Sox11 acting to promote neuronal differentiation. In addition, compared to control cultures, the maturity of transfected cells depended upon Sox11; at 2DIV, the length of a neuron's longest neurite tended to be longer in Sox11GOF and shorter in Sox11LOF neurons than control transfected cells (Fig. 2E,F). In contrast, at 2DIV, levels of Sox11 did not affect the number of primary neurites (Fig. 2G). By 5DIV staining with the axon-specific antisera SMI-312 revealed that Sox11GOF neurons had longer axons, while Sox11LOF neurons had shorter axons compared with control transfected cells (Fig. 2H,I).

**Fig. 3.**
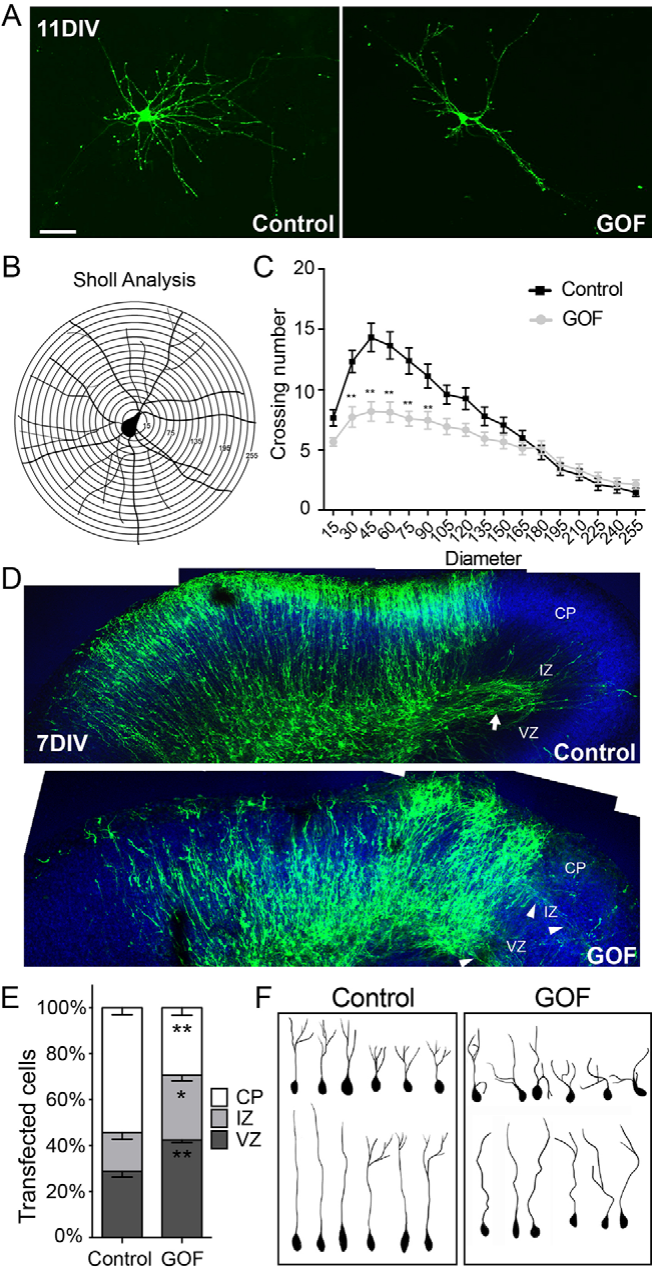
**Misexpression of Sox11 in mouse cortical neurons interferes with dendritic extent *in vitro* and localization and shape of neurons *in vivo*.** (A-C) Images of primary cortical neurons expressing GFP alone (left) or GFP and Sox11 (right) (A). A schematic of Sholl analysis of a cortical neuron in which neurite crossings for each ring are quantified (B). Overexpression of Sox11 (gray) reduced the complexity of dendritic branching of cortical neurons *in vitro* compared to control neurons (black) (C). (D) Organotypic slice cultures of cortex transfected at E14.5 with GFP alone (top) or GFP and Sox11 (bottom) and imaged at 7DIV. GFP^+^ cells have extended an axon tract in control (arrow) while in Sox11GOF, axons were diffuse (arrowheads). (E) Distribution of GFP^+^ cells in cortical embryonic zones is different in Sox11GOF compared to control; Sox11GOF cells tend to remain closer to the lateral ventricle, in the ventricular zone (VZ) and intermediate zone (IZ), than control-transfected cells. (F) Representative traces of neurons from control (left) and Sox11GOF (right) cortical plate (CP) reveals that neurons are less orderly when Sox11 expression is maintained. Scale bar: 30 μm (A); 63 μm (D). Data represented as mean±sem; *P<0.05; **P<0.01.

Previous expression data demonstrated that Sox11 levels decrease as mouse corticogenesis proceeds. To examine the consequences of elevated and protracted Sox11 expression, Sox11 GOF was examined in 11DIV cortical neuronal cultures (Fig. 3A) using Sholl analysis to assess the complexity of neuronal morphology (Fig. 3B). This analysis revealed that long-term elevation of Sox11 resulted in decreased dendritic branching and complexity compared to control neurons (Fig. 3C), indicating that tight regulation is critical for Sox11's function.

Morphological changes associated with extended Sox11 expression were also observed in organotypic slice cultures, a paradigm capable of maintaining the anatomical organization of the cerebral cortex *in vitro*. In this experimental approach, embryonic cerebral cortex were transfected with control or Sox11GOF vectors using EUE and the resultant brains were sliced, with the slices maintained in culture (Fig. 3D). After 7DIV, which is equivalent to P1 or P2 *in vivo*, there was extensive evidence of transfected cells throughout the cerebral wall, including considerable staining within the cortical plate and robust axonal projections present in control-transfected slices (Fig. 3D,E). In contrast, elevated and prolonged Sox11 expression resulted in a greater proportion of transfected cells in the ventricular and intermediate zones, fewer transfected cells within the cortical plate, and fewer labeled axonal projections (Fig. 3D,E). In addition, analysis of neuronal morphology for cells within the cortical plate revealed that dendritic branching was generally more disorganized in Sox11GOF than control-transfected cells (Fig. 3F). Together, these data demonstrate that elevated and sustained Sox11 expression interferes with proper neuronal maturation, with potential deficits in both migration and differentiation.

### Sox11 in frog neurogenesis

**Fig. 4.**
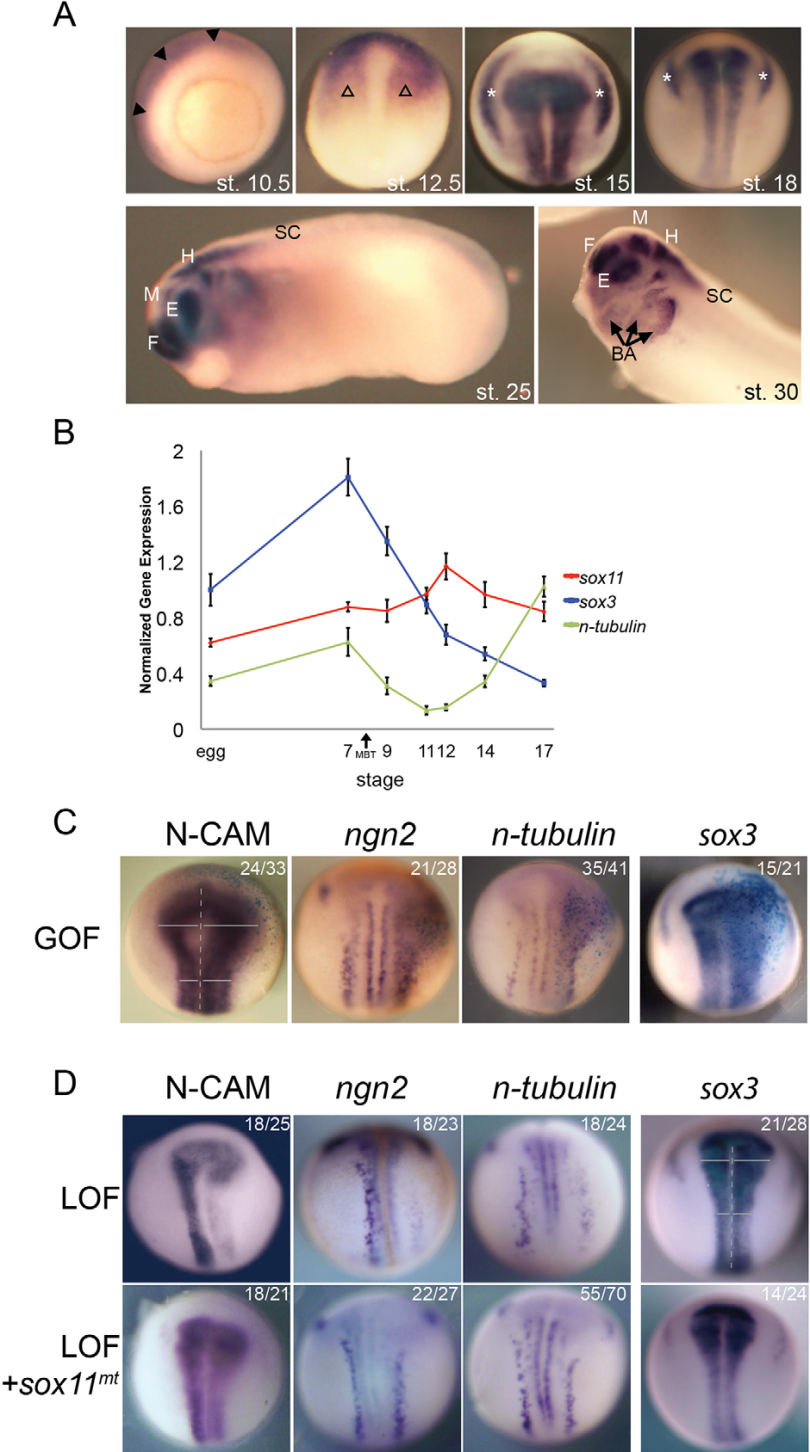
***Sox11* is expressed in neural tissue and influences neural development in *Xenopus laevis*.** (A) Whole mount *in situ* hybridization (WISH) of *sox11* in frog embryos at st. 10.5 (vegetal view), 12.5, 15 and 18 (dorsal view along anterior-posterior axis), 25 (lateral view, with head to the left), 30 (lateral view of head) reveals *sox11* is expressed in the presumptive CNS. Filled arrowhead marks ectoderm. Open arrowhead marks anterior neural plate. Asterisk marks intermediate and posterior placodes. Arrow marks branchial arches. F, forebrain; M, midbrain; H, hindbrain; E, eye; SC, spinal cord; BA, branchial arches. (B) Quantitative PCR of *sox3*, *n-tubulin* and *sox11* expression during frog development reveals *sox11* is expressed throughout early embryonic development. Data represented as mean±sem. (C-D) WISH of st. 15 embryos injected on the right side with *sox11* RNA (dorsal view, along the anterior-posterior axis). *sox11*GOF expands expression of pan-neural marker, N-CAM, the proneural gene, *ngn2*, the neuronal marker, *n-tubulin*, and the neural progenitor marker, *sox3*. (D) WISH of st. 15 embryos injected on the right side with Sox11 morpholinos alone (top) or Sox11 morpholinos and *sox11^mt^* RNA (bottom). When Sox11 is reduced, expressions of N-CAM, *ngn2* and *n-tubulin* were decreased, and expression of *sox3* was increased; all LOF phenotypes were reversed when *sox11^mt^* was expressed.

To characterize the expression of *sox11* in frog neural development, whole mount *in situ* hybridization (WISH) of *Xenopus laevis* embryos at discrete stages was performed (Fig. 4A). At the early gastrula stage when neural tissue is newly specified [stage (st.) 10.5], *sox11* is expressed throughout the ectoderm. By the end of gastrulation (st. 12.5), *sox11* is expressed primarily in the anterior neural plate. In the mid-to-late neurula stages (st. 15 and st. 18), expression is also detected in the neural tube and intermediate and posterior placodes. In the tail bud stages (st. 25 and st. 30), when the brain is segmented into distinct regions, *sox11* continues to be strongly expressed in the forebrain, midbrain and hindbrain, the eye and branchial arches but is at low levels in the spinal cord. In the tadpole stage, *sox11* continued to be expressed at high levels in the brain and the posterior the spinal cord (data not shown). This expression pattern of frog *sox11* is consistent with previous studies (Yanai et al., 2011; Cizelsky et al., 2013; Uy et al., 2015).

To chart levels of expression over developmental time, quantitative RT-PCR was used (Fig. 4B). *sox11* expression was compared to expression of markers of progenitor cells, *sox3*, and of neurons, *n-tubulin*. *sox3* is maternally deposited and decreases over time as progenitors transition to differentiated cells. In contrast, *n-tubulin*, a marker of neurons, increases at st.14, continues to rise, and peaks at st. 17 or later as neuronal differentiation occurs (Fig. 4B). On this backdrop, *sox11* levels are high early in development, peak at the end of gastrulation (st. 12) and then decrease.

We next sought to characterize Sox11's function during frog primary neurogenesis using GOF and LOF approaches. To this end, one cell of a two-cell *Xenopus* embryo was injected with *sox11*GOF RNA or a *sox11*LOF morpholino (MO) and resultant embryos were examined at later stages. Overexpression of *sox11* expanded expression of the pan neural marker N-CAM, the proneural gene *neurogenin2* (*ngn2*), and the neuronal marker *n-tubulin*, consistent with Sox11 promoting neural differentiation (Fig. 4C). Surprisingly, *sox3*, a marker of neural progenitors, was also expanded when *sox11* was overexpressed; a result that is consistent with the idea that Sox11 promotes both neural induction and neuronal differentiation in frogs (Fig. 4C) (Yan et al., 2009b).

Results of LOF analyses revealed that expression of neural markers, N-CAM, *ngn2*, and *n-tubulin* were all decreased while *sox3* was slightly expanded, consistent with impairment of neuronal differentiation in Sox11 morphant embryos (Fig. 4D). To confirm that LOF phenotypes were specific for Sox11, rescue experiments using a MO-insensitive form of *sox11* (*sox11^mt^*) were performed (Fig. S2). *sox11^mt^* RNA rescued the shifts in *ngn2*, N-CAM, *n-tubulin*, and *sox3* expression that were observed in the LOF paradigm.

### Redundancy of mouse and frog Sox11 function

**Fig. 5.**
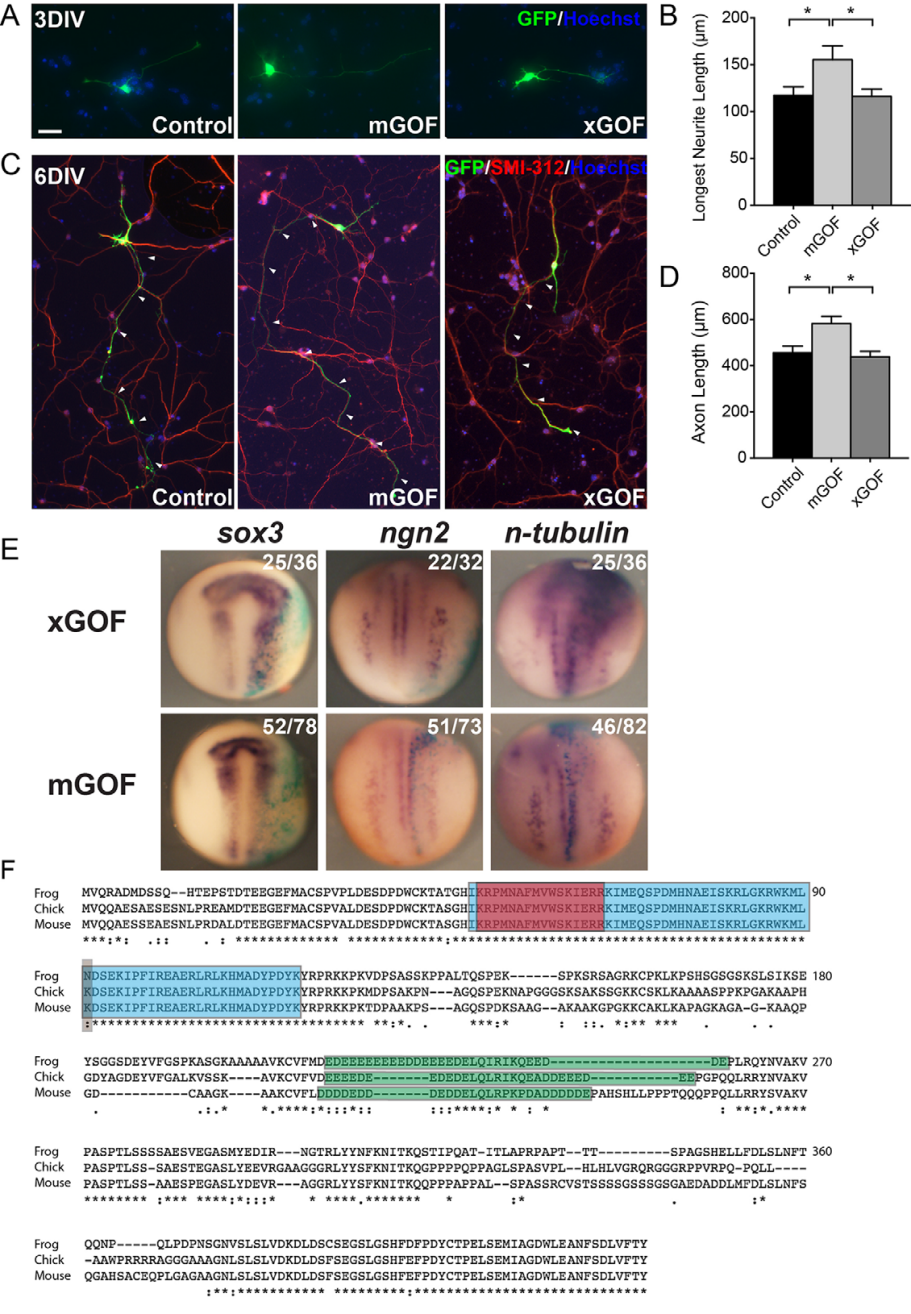
**Functional reciprocity of Sox11 between frog and mouse.** (A-D) Primary cortical neurons expressing GFP alone (left), GFP and mouse Sox11 (mGOF, middle), and GFP and *Xenopus sox11* (xGOF, right) and analyzed at 3DIV (A,B) and 6DIV (C,D) revealed increases in the longest neurite (B) and axon (D) when mouse Sox11 but not frog *sox11* was expressed. Data represented as mean±sem; *P<0.05. (E) WISH of st. 15 embryos (dorsal view, along with the anterior-posterior axis) injected at the two-cell stage with either frog (top) or mouse (bottom) *sox11* and *lacZ* tracer (blue). xGOF elevated levels of *sox3*, *ngn2* and *n-tubulin*, while mGOF had no effect. (F) Alignment of frog, chicken and mouse Sox11 protein sequences. Frog sequence shown here is Sox11a sequence: Sox11a and Sox11b share 93% similarity, and the single amino acid difference observed between frog Sox11a and other species is also present in Sox11b sequence. Discrete protein domains are shaded: blue is the HMG box, red is the nuclear localization signal; green is the acid-rich region; grey marks the one amino acid difference in the HMG box. (*=Identical amino acid residues; :=Different but highly conserved amino acid residues;∙=Different but somewhat similar amino acid residues; blank=dissimilar amino acid residues or gap.) Scale bar: 32 μm (A); 84 μm (C).

Taken together, results from mouse and frog indicate that Sox11 is a regulator of neuronal maturation. To determine whether functional redundancy exists between mouse and frog Sox11, mouse cortical cultures were transfected with the *Xenopus laevis sox11* (xSox11) GOF vector (Fig. 5A,C). Unlike transfection of mouse cortical neurons with *mus musculus* Sox11 (mSox11), which resulted in both longer neurites at 3DIV and longer axons at 6DIV, mouse cortical cells transfected with xSox11GOF were no different from control-transfected neurons (Fig. 5B,D). Similarly, while injection of *Xenopus* embryos with *xsox11* mRNA resulted in expanded expression of *sox3*, *ngn2*, and *n-tubulin* at st. 15, there was no change in the expression of these genes when *mSox11* mRNA was expressed in frog embryos (Fig. 5E). Thus, mouse and frog Sox11 are not functionally interchangeable in these assays.

To understand differences in mouse and frog Sox11, a bioinformatics comparison of the Sox11 protein sequences of mouse, frog, and the evolutionary intermediate, chick, was performed (Fig. 5F). This analysis revealed that the frog sequence is 69.81% identical to chick and 68.18% identical to mouse, while the chick sequence is 75.8% identical to mouse. Although there is considerable difference in sequence throughout the proteins, it is notable that there is near perfect conservation within the HMG domain between species. One exception exists, however, a single amino acid change in the HMG domain; the basic, polar amino acid lysine in chick and mouse Sox11 is substituted for with asparagine, a neutral, polar amino acid, in both frog Sox11a and b. This difference in sequence might explain the lack of functional redundancy between mouse and frog.

## DISCUSSION

The formation of a functional nervous system requires coordinated regulation of gene expression in order to designate cellular identity. While molecules that support specific functions, including patterning of the early nervous system, control of cell proliferation within germinal zones, regulation of differentiation of neurons and glia, have been identified, molecular control of transitions from one state to another, such as the shift of a progenitor cell into a neuron, require examination. Previous data suggest that Sox11 is involved in neuronal differentiation (Bergsland et al., 2006, 2011; Mu et al., 2012; Cizelsky et al., 2013; Chen et al., 2015; Uy et al., 2015), but these data did not indicate at which stage in neural development Sox11 acts. Furthermore, comparison of Sox11 function between evolutionarily discrete nervous systems, so as to identify similarities and differences between species, had not been undertaken. Thus, characterizing the molecules that underlie cellular transitions in the nervous system, particularly the role of transcriptional regulators, such as Sox proteins, is a critical undertaking.

Our results demonstrate that during mouse cerebral cortical development Sox11 is expressed within embryonic compartments that contain differentiated neurons*.* In addition to the anticipated pro-differentiation role for Sox11, GOF and LOF studies reveal a novel function: promotion of morphological complexity of both axons and dendrites. Results from these studies also highlighted the importance of the time-dependent activation and inactivation observed in cortical neurons; when Sox11 expression is artificially elevated and maintained, neuronal morphology is significantly more simple compared with wild type neurons in which Sox11 has been inactivated. Thus, in mouse cerebral cortex, Sox11 promotes neuronal differentiation and influences neuronal shape in a level- and time-dependent manner. In frog, *sox11* is maternally encoded initially, is highly expressed in developing neural tube, and then broadly present in the tadpole brain. Given this complex expression pattern, the relative roles of maternal versus zygotic Sox11 will be investigated in our future studies.

While, as anticipated, data from GOF and LOF studies support a role for Sox11 in promoting neuronal differentiation in both mouse and frog, Sox11 unexpectedly seems to also affect the induction or maintenance of neural progenitors by elevating *sox3* expression in frog. This action may be a consequence of the unique development of *Xenopus*; Sox3 is initially maternally expressed and localized to the animal pole. It is proposed that Sox3 functions to restrict β-catenin activity and therefore the expression of genes required for dorsalization (Zorn et al., 1999). At the same time, Sox11 may affect β-catenin activity directly, since when Sox11 binds xNLK TCFs bound to β-catenin are phosphorylated, thus suppressing the DNA-binding activity of the TCF-4/β-catenin complex and activation of target genes (Hyodo-Miura et al., 2002). Taken together, Sox11 seems likely to affect the β-catenin signaling pathway in regulating neuronal development in frog.

How might a single transcription factor control both the expansion of dividing precursors and the production of post-mitotic neurons? A possibility is that Sox11 acts as both an activator and a repressor functions, based upon partner protein interaction and target gene identity. For example, Sox11 has been shown as a direct activator for Tubb3 (Dy et al., 2008; Chen et al., 2015), however, when Sox11 partners with Brn2 there is suppression for Tubb3 (Chen et al., 2015). Together, this partner-specific transcriptional activity may explain unanticipated roles for Sox11 in both mouse and frog.

Unusually, mouse and frog Sox11 did not mimic one another in GOF studies involving the expression of one species’ Sox11 in the other species’ assay system (i.e. developing embryos for frogs and cultures of cortical neurons for mice.) Comparison of mouse and frog Sox11 revealed extensive sequence differences between these two orthologs. While there is significant sequence divergence throughout the extent of these proteins, a single amino acid change in the HMG domain between mouse and frog Sox11 is notable, as the HMG motif is usually tightly conserved between species (Mollaaghababa and Pavan, 2003). It is possible that this single amino acid difference at position 91 – in mouse, the basic residue lysine and in frog, the neutral amino acid asparagine – may be responsible for functional differences. Taken together, sequence differences between mouse and frog Sox11 may explain the lack of functional reciprocity between these proteins.

The formation of the mouse cerebral cortex epitomizes the complexity of nervous system development, while the creation of the embryonic frog brain represents a more straightforward process. Our data demonstrate that Sox11 acts in both these developmental processes by promoting neuronal differentiation. Other roles were also revealed; mouse Sox11 controls neuronal morphology and frog Sox11 enhances neural progenitor pool expansion. These findings are consistent with previous studies that show that mouse Sox11 regulates axonal growth of embryonic sensory neurons and that frog Sox11 acts with FoxD5 to maintain the neural plate in a proliferative state in *Xenopus* (Yan et al., 2009a,b; Lin et al., 2011). On a molecular level, our result demonstrates that frog Sox11 acts upstream of the proneural protein Ngn2, a key regulator of commitment of progenitors to a neuronal fate. This is in contrast to Sox11 in the chick spinal cord, which acts downstream of this same regulator of neuronal specification (Bergsland et al., 2006). Similar to chick, mouse Sox11 expression is also preceded by Ngn2 expression (Chen et al., 2015); however, whether the two proteins act in the same pathway in this organism is unknown. Our data illustrate that between frog, chick, and mouse, Sox11 shows differences in activity in the context of Ngn2, suggesting that for different organisms Sox11 acts at distinct times in the time course of neuronal development. Ultimately, by shedding light on the similarities and differences in Sox11 between mouse and frog, our investigation offers insight into the potential roles of Sox11, and how those roles changed over the course of evolution.

## MATERIAL AND METHODS

### Animal usage

All mouse and frog use and care was in accordance with federal and institutional guidelines, particularly Georgetown University's Institutional Animal Care and Use Committee protocols 12-018-100035 and 13-016-100085, respectively. To obtain mouse cortical tissue samples and cells, timed CD1 wild type pregnant females mice were euthanized. Brains or cerebral cortex of embryos, presumably equal amounts of both sexes, were dissected and either dissociated for cell culture or fixed, frozen, and sectioned for staining. *Xenopus laevis* embryos were obtained using standard methods (Sive et al., 2000) and staged (Nieuwkoop and Faber, 1994).

### RT-PCR

For analysis of mouse Sox11 expression, total RNA was isolated from cerebral cortical tissue of embryonic day (E)10.5, 12.5, 14.5, 16.5, 18.5, and postnatal day (P)0, 5 and 10 mice (*in vivo*) or differentiated cortical cultures at days *in vitro* (DIV)1, 2, 3, 5, 8, 10 and 13 using the Tri-Reagent Kit (Sigma). cDNA was synthesized using the First Strand cDNA Synthesis Kit (Invitrogen). Primers specific to Sox11 and U6, as an internal control, were used for endpoint PCR. For frog, total RNA was isolated from egg or embryos at egg, or stage (st.) 7, 9, 11, 12, 14 and 17 using TRIzol^®^ Reagent (Life technologies) as previously described (Amin et al., 2014). cDNA was synthesized using random hexamers and TetrocDNA Synthesis Kit (Bioline). qPCR was performed with SensiFAST™ SYBR^®^ No-ROX Kit (Bioline). Using *sox11* forward Primer: 5′–TAAGGACCTGGATTCCTTCAGCGA–3′; *sox11* reverse Primer: 5′–TCAATACGTGAACACCAGGTCGGA–3′, levels of *sox11*, *sox3* (Whittington et al., 2015), and *n-tubulin* (Klisch et al., 2006) expression were normalized to amplification of the internal control, ornithine decarboxylase (ODC) (Kiyota et al., 2008). For analysis of *sox11*, expression in *Xenopus sox11a* and *b*, which are 93% identical, was assessed. All samples were analyzed in triplicate and experiments were repeated at least twice.

### Immunohistochemistry

Immunohistochemistry was performed with sections mounted on microscope slides or neurons on coverslips as previously described (Clifford et al., 2014). Samples were incubated with blocking solution (5% donkey serum, 0.1% lysine, 1% glycine, 1% BSA, 0.4% Triton X-100 in PBS) for 1 h at room temperature and then incubated with the following primary antibodies and dilutions: anti-Sox11 (1:1500, from Sock laboratory; Hoser et al., 2008), anti-Tubb3 (1:1000, Covance), anti-SMI-312 (1:500, Covance) and with Hoechst stain (1:10,000, Life Technologies) followed by washes and incubation with species-appropriate Alexa Fluor secondary antibody (Invitrogen).

### *In situ* hybridization

For mouse, E14.5, E17.5 and P10 samples were collected, and *in situ* hybridization for Sox11 (vector to generate mouse probe was a gift from Veronique Lefebvre) was performed as previously described (Clifford et al., 2014). For frog, whole mount *in situ* hybridization (WISH) was performed as previously described (Hemmati-Brivanlou et al., 1990; Harland, 1991) with these changes: length of pre-hybridization step was increased to overnight and the RNAse treatment step was eliminated. Digoxygenin labeled mRNA probes were generated for *sox3* (Penzel et al., 1997), *ngn2* (Ma et al., 1996), *n-tubulin* (Richter et al., 1988), *sox11* and N-CAM (Kintner and Melton, 1987) from plasmids produced by the Silva Laboratory (Georgetown University).

### Function analyses

For mouse, expression vectors for mouse Sox11 GOF (gift from Veronique Lefebvre; Potzner et al., 2010) or shRNA constructs specific for Sox11 LOF (Chen et al., 2015) (1.5 μg/μl each) and CMV-eYFP (0.5 μg/μl) were delivered to the dorsal cortex via *ex utero* electroporation (Chen et al., 2015). Cultures of differentiated cortical neurons or organotypic slices were created as previously described (De Simoni and Yu, 2006; Chen et al., 2015). Upon harvest, samples were fixed with 4% paraformaldehyde for immunocytochemistry. YFP^+^ transfected cells were imaged, and neurite and axon lengths were measured. 45-60 neurons or 4-6 slices from three experiments were analyzed for each condition.

For frog, embryos were injected with *in vitro* synthesized capped mRNAs using mMESSAGE mMACHINE^®^ Transcription Kit (Life Technologies). The mutation in the *sox11^mt^* plasmid was generated as described (Braman et al., 1996) with primers listed in Table S1. Morpholinos were confirmed to block *sox11* in the *in vitro* translation assay using TNT^®^ SP6 High-Yield Wheat Germ Protein Expression System (Promega) (Fig. S2). Briefly, 1 μg of mRNA was incubated with or without indicated morpholinos and *sox11^mt^* RNA in the *in vitro* translation system at 30° for 2 h, proteins was denatured and separated on Tris-glycine polyacrylamide gel. The morpholino blocked wild type but not *sox11^mt^* protein translation. Either 1200 pg *sox11* RNA and 300 pg *lacZ* RNA (tracer) or 60 ng Sox11 morpholino1 and Sox11 morpholino2 (GENE TOOLS, LLC) were injected into one cell of a two-cell stage embryo to over-express or knock down Sox11. 1200 pg *sox11^mt^* RNA was injected to rescue morphant embryos. Embryos were cultured until neurula stage.

For cross-species experiment, mouse or frog Sox11 expression plasmids were transfected into HEK293 cells, expression was confirmed by immunohistochemistry using anti-Sox11 antibody [anti-mouse Sox11, 1:1500, gift from Sock laboratory (Hoser et al., 2008) or anti-frog Sox11, 1:500, Everest Biotech].
